# Trained Attendants and Nurses for the Insane

**Published:** 1891-06-20

**Authors:** 


### TRAINED ATTENDANTS AND NURSES FOR THE INSANE.

At the May examination for the Certificate of Proficiency in Nursing and Attending on the Insane, held by the Medico-Psychological Association of Great Britain and Ireland, all the candidates from the James Murray's Royal Asylum, Perth, passed successfully, viz.:—Nurses Jamieson, Scott; and Attendants Knight, Smith, and Pennycook.

The requirement of this examination are, at least two years' Asylum training, and the passing of an examination (partly written, partly oral) on the special duties of nurses and attendants, including a knowledge of the elements of anatomy and physiology.

